# Hyperinflammatory Syndrome, Natural Killer Cell Function, and Genetic Polymorphisms in the Pathogenesis of Severe Dengue^[Author-notes jiac093-FM1]^

**DOI:** 10.1093/infdis/jiac093

**Published:** 2022-03-10

**Authors:** Nguyen Lam Vuong, Ka Wai Cheung, Balamurugan Periaswamy, Tran Thuy Vi, Huynh Thi Le Duyen, Yan Shan Leong, Zayanah Noor Binte Hamis, Michaela Gregorova, Eng Eong Ooi, October Sessions, Laura Rivino, Sophie Yacoub

**Affiliations:** Oxford University Clinical Research Unit, Ho Chi Minh City, Vietnam; University of Medicine and Pharmacy at Ho Chi Minh City, Ho Chi Minh City, Vietnam; Duke–National University of Singapore Medical School, Singapore; Genome Institute of Singapore, Singapore; Oxford University Clinical Research Unit, Ho Chi Minh City, Vietnam; Oxford University Clinical Research Unit, Ho Chi Minh City, Vietnam; Duke–National University of Singapore Medical School, Singapore; Duke–National University of Singapore Medical School, Singapore; School of Cellular and Molecular Medicine, University of Bristol, Bristol, United Kingdom; Duke–National University of Singapore Medical School, Singapore; Saw Swee Hock School of Public Health, National University of Singapore, Singapore; Duke–National University of Singapore Medical School, Singapore; Saw Swee Hock School of Public Health, National University of Singapore, Singapore; Department of Pharmacy, National University of Singapore, Singapore; Duke–National University of Singapore Medical School, Singapore; School of Cellular and Molecular Medicine, University of Bristol, Bristol, United Kingdom; Oxford University Clinical Research Unit, Ho Chi Minh City, Vietnam; Centre for Tropical Medicine and Global Health, University of Oxford, Oxford, United Kingdom

**Keywords:** dengue, hyperinflammation, macrophage activation syndrome, single-nucleotide polymorphisms, immunomodulation

## Abstract

**Background:**

Severe dengue, characterized by shock and organ dysfunction, is driven by an excessive host immune response. We investigated the role of hyperinflammation in dengue pathogenesis.

**Methods:**

Patients recruited into an observational study were divided into 3 plasma leak severity grades. Hyperinflammatory biomarkers were measured at 4 time points. Frequencies, activation, and cytotoxic potential of natural killer (NK) cells were analyzed by flow cytometry. RNA was extracted from sorted CD56^+^ NK cells and libraries were prepared using SMART-Seq and sequenced using HiSeq3000 (Illumina).

**Results:**

Sixty-nine patients were included (grade 0, 42 patients; grade 1, 19 patients; grade 2, 8 patients). Patients with grade 2 leakage had higher biomarkers than grade 0, including higher peak ferritin levels (83.3% vs 45.2%) and H-scores (median, 148.5 vs 105.5). NK cells from grade 2 patients exhibited decreased expression of perforin and granzyme B and activation markers. RNA sequencing revealed 3 single-nucleotide polymorphisms in NK cell functional genes associated with more severe leakage—NK cell lectin-like receptor K1 gene (*KLRK1*) and perforin 1 (*PRF1*).

**Conclusions:**

Features of hyperinflammation are associated with dengue severity, including higher biomarkers, impaired NK cell function, and polymorphisms in NK cell cytolytic function genes (*KLRK1* and *PRF1*). Trials of immunomodulatory therapy in these patients is now warranted.

## Teaser

Patients with severe dengue have higher inflammatory biomarkers, impaired natural killer (NK) cell activation/cytotoxic potential, and polymorphisms in genes involved in NK cell cytolytic function.

Dengue causes a substantial global public health burden, with recent estimates suggesting that 51–96 million symptomatic infections occur each year in >120 countries [1]. Dengue presents as a spectrum of clinical syndromes from a nonspecific febrile illness to severe dengue, characterized by increased capillary permeability, which can lead to hypovolemic shock, coagulopathy, and organ impairment [2]. There are currently no licensed antiviral or adjunctive therapeutics. The pathogenesis of dengue is multifactorial; however, an immune-mediated pathogenesis likely plays a key role with hypercytokinemia and an excessive host inflammatory response associated with life-threatening complications [3–5]. Monocytes and macrophages are key dengue viral target cells and both antibody-dependent enhancement as well as dysfunctional cytotoxic T-cell/natural killer (NK) cell responses facilitate increased uptake/replication and decreased clearance of these cells, resulting in higher viral loads and more severe disease [4, 6].

Cytokine storm syndrome encompasses a number of clinical syndromes associated with hyperinflammation including macrophage activation syndrome (MAS), secondary hemophagocytic lymphohistiocytosis (HLH), and macrophage activation–like syndromes [7, 8]. The clinical features include fever, organ dysfunction, cytopenias, vascular leakage, bleeding, and hyperferritinemia [9]. Hyperinflammation is likely to be an underrecognized phenomenon in severe dengue, with which there is considerable overlap in diagnostic criteria (eg organ dysfunction, bleeding, and vascular leakage) [10]. The underlying mechanism of MAS/hyperinflammation includes a reduced NK/CD8 T-cell functional capacity to lyse infected macrophages/monocytes, leading to uncontrolled macrophage expansion, augmented viral replication, and release of proinflammatory cytokines, resulting in end-organ damage [11]. Several genetic mutations in the NK cell cytolysis pathway have been associated with this phenotypic dysfunction of NK cell activity in these hyperinflammatory diseases [12].

Markers of MAS/hyperinflammation including high levels of ferritin, triglycerides, and soluble CD163 (sCD163) have been demonstrated in dengue and are associated with disease severity [13, 14]. The similarity in clinical and laboratory features of MAS and severe dengue raises the possibility that they share some common pathways; however, many gaps in our knowledge remain, including NK cell function, genetic polymorphisms, and association with dengue disease severity. Correct recognition of features of MAS/hyperinflammation in dengue, where only supportive treatments are available, could have an immediate clinical impact, as hyperinflammation responds to specific immunomodulating therapies [15–17]. Anticytokine therapies such as interleukin (IL) 1 and IL-6 blockade have been shown to effectively treat many hyperinflammatory conditions, irrespective of initiating trigger, by reducing systemic inflammation and downstream tissue damage [18].

In this study, we test the hypothesis that markers of hyperinflammation/MAS are associated with dengue disease severity, including specific MAS biomarkers, NK cell activation, and cytotoxic potential and polymorphisms in NK cell genes.

## MATERIALS AND METHODS

### Clinical Cohort

Patient samples and clinical information were included from all the patients recruited into an observational study conducted in Vietnam between 2013 and 2016; the full study protocol has been published elsewhere [19]. In brief, this was a prospective observational study of adults and children >5 years of age presenting to the National Hospital for Tropical Diseases (Hanoi, Vietnam). Patients who were hospitalized with a diagnosis of dengue were eligible for recruitment. Patients were defined as having laboratory-confirmed dengue if reverse-transcription polymerase chain reaction, NS1 antigen, or dengue virus immunoglobulin M (IgM) assay was positive at enrollment, or if there was IgM seroconversion between paired serum samples. Participants were followed daily until resolution of their acute illness. Research blood samples were obtained at 4 time points: enrollment, 48 hours later, hospital discharge/days 5–7 and follow-up between days 14 and 21 of illness. Patients had plasma stored as well as peripheral blood mononuclear cells (PBMCs) separated and stored in liquid nitrogen. Clinical end-point was plasma leakage, which was subdivided into 3 grades: no leakage (grade 0), moderate leakage (grade 1), and severe leakage (grade 2) (Supplementary Appendix 1). The H-score was calculated using the criteria developed by Fardet et al excluding the requirement for bone marrow aspirate [20].

Ethical approval was obtained from the Oxford Tropical Research Ethics Committee and the Ethics Review Committee at the National Hospital for Tropical Diseases. Written informed consent was obtained from all participants. Experimental work on PBMCs was performed at Duke–National University of Singapore (NUS) Medical School (Singapore) after approval by the Singapore-NUS Institutional Review Board.

### Biomarkers

MAS biomarkers, including ferritin, sCD163, soluble IL-2Ra (soluble CD25 [sCD25]), triglycerides, and fibrinogen, as well as IL-18 and IL-1Ra, were measured at 4 time points: enrollment, 48 hours later, hospital discharge, and follow-up at 14 days after illness onset. These tests were performed using commercial enzyme-linked immunosorbent assay kits to measure fibrinogen (Abcam), triglyceride (Cayman), and IL-18 (R&D Systems) levels and magnetic bead–based assays (R&D Systems) on a Luminex 200 analyzer to evaluate ferritin, sCD163/sCD25, and IL-1Ra concentrations, according to the manufacturer’s specifications.

### NK Cell Phenotyping

The PBMC preparation is shown in Supplementary Appendix 1. Antibodies used are listed in Supplementary Table 1 and gating strategy in Supplementary Figure 1. We concatenated the flow cytometry standard (FCS) files containing the data from NK cell populations and performed a *t*-distributed stochastic neighbor embedding (tSNE) analysis on live CD14^–^CD3^–^ cells. tSNE is a dimensionality reduction algorithm that allows visualization of multidimensional data into 2 dimensions by mapping single cells based on the relative distribution of all markers analyzed, with cells displaying similar marker expression mapped in close proximity [21]. For the tSNE analysis, FCS files from 50 patients were concatenated after downsampling and tSNE analysis was done by using a plugin in FlowJo version 10.8.0 software. Calculations were performed via Barnes–Hutt algorithm with 1000 iterations, a perplexity of 30, and learning rate 4959.

### RNA Sequencing and Single-Nucleotide Polymorphism Analysis

The quality and quantity of the RNA extracted from the sorted CD56^+^ NK cells obtained from 68 patients were assessed by Agilent 2100 bioanalyzer. The libraries of the samples were prepared from 100 pg RNA using SMART-Seq Stranded Kit. The samples were sequenced using HiSeq3000 (Illumina) to obtain 150-bp paired-end reads. Raw reads (fastq) were mapped to human GRCh38, release 97, reference using STAR aligner. Reads that were unambiguously aligned to genes/features were counted using HTSeq. Single-nucleotide polymorphisms (SNPs) were identified in the RNA-Seq data set using LoFreq2 call [22]. We assessed the association of known NK cell SNPs (Supplementary Table 2) as well as novel SNPs in these genes by dengue disease severity.

### Statistical Analysis

Clinical and laboratory data were analyzed using the statistical software R version 3.6.1. We summarized the data using median and interquartile range (IQR) for numeric variables, and number of cases and percentage for categorical variables. Biomarker variables were compared between the 3 severity groups (leakage grades 0, 1, and 2) by nonparametric Kruskal–Wallis test. In case of significant difference, pairwise comparisons were performed using Dunn multiple comparison test. All statistical tests were 2-sided. The biomarkers were summarized and illustrated by illness day groups (days 1–3, 4–5, 6–7, 8–10, and >10). For the NK cell phenotype data, statistical significance was determined by Kruskal–Wallis test followed by Dunn multiple comparison test using GraphPad Prism version 9.1c. *P* values < .05 were considered statistically significant. Statistical significance of SNPs between grades 0, 1, and 2 were defined by Fisher exact test.

## RESULTS

### Clinical and Biomarker Characteristics

A total of 69 patients hospitalized with laboratory-confirmed dengue were included in this study (42 with leakage grade 0, 19 with grade 1, and 8 with grade 2). The demographic and clinical characteristics of the cohort are shown in Table 1. Overall, 49.3% were female and the median age was 29 years. Median illness day at hospital admission was 6 (IQR, 5–7). There was a mixture of different dengue serotypes. Viral load was lower in grade 2 patients; however, they were enrolled slightly later in their illness course, so are not comparable (Table 1). Most patients had a decreased platelet count (median, 58 [IQR, 33–95] × 10^9^/L). Classification according to the 2009 World Health Organization guidelines resulted in 5 (7.2%) patients with severe dengue and 46 (66.7%) patients with dengue with warning signs.

**Table 1.** **Clinical Characteristics at Hospital Admission, by Disease Severit**y

Characteristic	No.	All Patients (N = 69)	No.	Grade 0 (n = 42)	No.	Grade 1 (n = 19)	No.	Grade 2 (n = 8)
Age, y	69	29.0 (22.0–36.0)	42	29.0 (22.2–36.8)	19	29.0 (21.5–36.0)	8	23.5 (18.2–28.0)
Sex, female, No.	69	34 (49.3)	42	19 (45.2)	19	10 (52.6)	8	5 (62.5)
Illness day at enrollment	69	6.0 (5.0–7.0)	42	6.0 (5.0–7.0)	19	5.0 (4.5–6.0)	8	6.0 (5.8–7.2)
Serotype, No.	69		42		19		8	
DENV-1		12 (17.4)		7 (16.7)		4 (21.1)		1 (12.5)
DENV-2		10 (14.5)		6 (14.3)		3 (15.8)		1 (12.5)
DENV-3		16 (23.2)		10 (23.8)		6 (31.6)		0 (0.0)
DENV-4		12 (17.4)		7 (16.7)		3 (15.8)		2 (25.0)
Unknown		19 (27.5)		12 (28.6)		3 (15.8)		4 (50.0)
Immunostatus, No. (%)	69		42		19		8	
Probable primary infection		7 (10.1)		3 (7.1)		3 (15.8)		1 (12.5)
Probable secondary infection		35 (50.7)		21 (50.0)		10 (52.6)		4 (50.0)
Inconclusive		27 (39.1)		18 (42.9)		6 (31.6)		3 (37.5)
Hepatomegaly, No. (%)	68	5 (7.4)	42	2 (4.8)	18	1 (5.6)	8	2 (25.0)
Platelets, ×10^9^/L	69	58.0 (33.0–95.0)	42	53.5 (33.0–105.0)	19	64.0 (38.5–91.0)	8	49.0 (31.5–68.2)
White cell count, ×10^9^/L	69	3.6 (2.4–4.7)	42	3.6 (2.5–4.7)	19	2.7 (1.6–4.0)	8	5.5 (3.6–7.5)
Neutrophils, ×10^9^/L	69	1.4 (0.8–2.3)	42	1.3 (0.8–2.2)	19	1.3 (0.7–2.1)	8	1.9 (1.0–3.4)
Lymphocytes, ×10^9^/L	69	1.0 (0.7–1.9)	42	1.1 (0.8–1.7)	19	0.8 (0.6–1.5)	8	2.0 (1.5–4.4)
ALT, IU/L	37	34.0 (27.0–76.0)	21	34.0 (20.0–58.0)	11	47.0 (31.5–116.0)	5	61.0 (34.0–81.0)
Viremia^a^, log_10_ copies/mL	68	5.2 (0.0–6.9)	41	4.9 (0.0–6.3)	19	6.6 (5.0–7.6)	8	2.6 (0.0–5.9)
WHO classification, No. (%)	69		42		19		8	
Uncomplicated dengue		18 (26.1)		16 (38.1)		2 (10.5)		0 (0.0)
Dengue with warning signs		46 (66.7)		26 (61.9)		16 (84.2)		4 (50.0)
Severe dengue		5 (7.2)		0 (0.0)		1 (5.3)		4 (50.0)

Data are presented as median (interquartile range) unless otherwise indicated.

Abbreviations: ALT, alanine aminotransferase; DENV, dengue virus; WHO, World Health Organization.

There were 18 patients with undetectable viremia at enrollment; all were set as 0 in the log_10_ scale.

**Figure 1.**
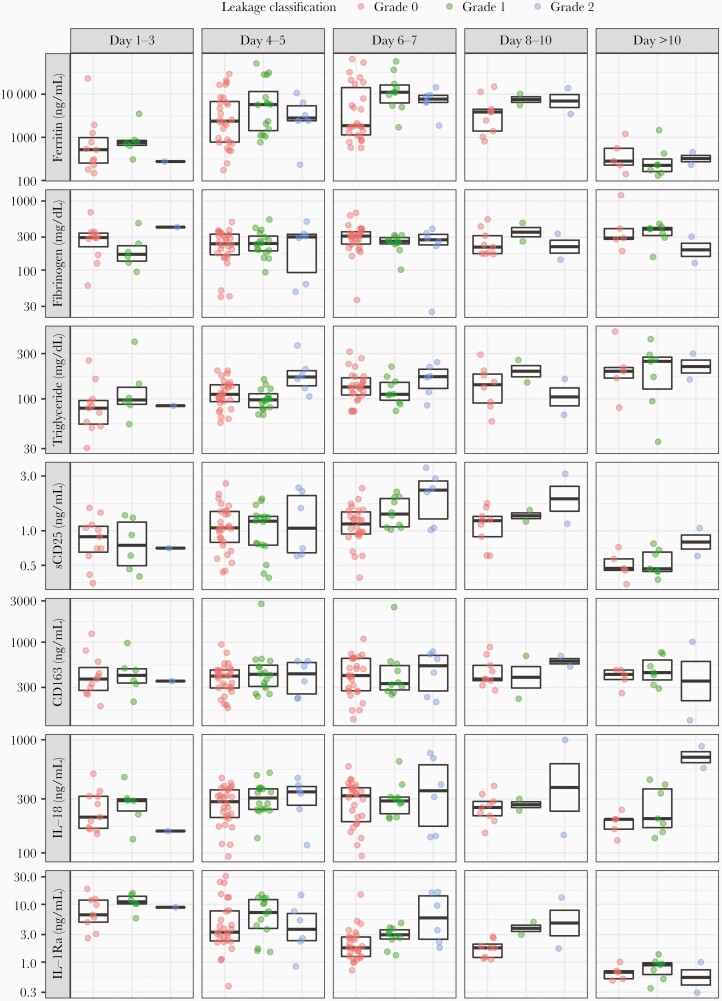
Kinetics of biomarkers over illness course, by disease severity. The middle line in each box represents the median. The upper and lower edges of each box represent the interquartile range. The points are the actual values of the biomarker levels and are colored by leakage grade. The y-axis is transformed using log-transformation. Abbreviations: IL, interleukin; sCD25, soluble CD25.

At hospital admission, patients with severe leakage (grade 2) had a median ferritin of 4809.1 (IQR, 2424.2–9832.6) ng/mL compared to patients without plasma leakage (grade 0), who had median levels of 1879.8 (IQR, 573.6–4696.4) ng/mL (Table 2). Patients with severe leakage also had higher triglycerides than patients without plasma leakage, and a higher proportion had ferritin levels >2000 ng/mL (83.3% vs 45.2%). The H-score for patients with severe leakage was significantly higher compared to those without: median H-scores of grades 0, 1, and 2 were 105.5 (IQR, 77.2–135.5), 138.0 (IQR, 107.5–167.0), and 148.5 (IQR, 114.5–177.2), respectively. Table 3 and Figure 1 show the kinetics of biomarkers over the illness course. In general, most increased during the first week of the illness course, except for fibrinogen with no clear trend and IL-1Ra with a decreased trend.

**Table 2.** Laboratory Characteristics, by Disease Severity

Characteristic	No.	Grade 0 (n = 42)	No.	Grade 1 (n = 19)	No.	Grade 2 (n = 8)	*P* Value^a^
Ferritin, ng/mL	41	1879.8 (573.6–4696.4)	19	2360.6 (831.0–10 528.3)	8	4809.1 (2424.2–9832.6)	.239
Fibrinogen, mg/dL	41	295.5 (198.1–349.0)	19	207.8 (153.7–266.7)	8	301.6 (250.8–356.6)	.152
Triglyceride, mg/dL	41	112.7 (77.9–142.5)	19	99.4 (84.0–127.0)	8	170.2 (126.9–208.3)	.037^b^
Soluble CD25, ng/mL	41	1.0 (0.7–1.3)	19	1.0 (0.7–1.3)	8	1.3 (0.7–2.2)	.497
Soluble CD163, ng/mL	41	374.7 (292.4–478.1)	19	416.5 (278.7–507.3)	8	485.2 (354.5–602.2)	.405
IL-18, ng/mL	41	309.0 (201.0–375.0)	19	291.0 (241.5–366.0)	8	346.5 (221.2–411.8)	.689
IL-1Ra, ng/mL	39	3.2 (2.3–7.1)	17	6.9 (3.7–11.8)	8	6.4 (2.2–9.1)	.198
Peak ferritin >2000 ng/mL, No. (%)	42	19 (45.2)	18	14 (77.8)	6	5 (83.3)	.027^c^
H-score	42	105.5 (77.2–135.5)	19	138.0 (107.5–167.0)	8	148.5 (114.5–177.2)	.013^c^
Features of MAS, No. (%)							
Fever ≥38.5°C	42	15 (35.7)	18	4 (22.2)	6	1 (16.7)	.498
2-line cytopenia	42	18 (42.9)	18	10 (55.6)	6	3 (50.0)	.695
Hepatomegaly and/or AST >100 U/L	42	6 (14.3)	18	5 (27.8)	6	2 (33.3)	.297
Ferritin >5000 ng/mL	42	13 (31.0)	18	13 (72.2)	6	3 (50.0)	.009^c^
Triglyceride >250 mg/dL or fibrinogen <150 mg/dL	42	8 (19.0)	18	4 (22.2)	6	2 (33.3)	.652

Data are presented as median (interquartile range) unless otherwise indicated.

Abbreviations: AST, aspartate aminotransferase; IL, interleukin; MAS, macrophage activation syndrome.

*P* values based on Kruskal–Wallis test.

Significant difference was shown in pairwise comparisons between grade 2 and grade 0 and between grade 2 and grade 1 (Dunn test).

Significant difference was shown in pairwise comparison between grade 1 and grade 0 (Dunn test).

**Table 3.** Kinetics of Biomarkers Over Illness Course

Biomarker	No.	Days 1–3 (n = 19)	No.	Days 4–5 (n = 50)	No.	Days 6–7 (n = 42)	No.	Days 8–10 (n = 13)	No.	Day >10 (n = 14)
Ferritin, ng/mL	18	587.3 (289.9–850.5)	50	2549.3 (1069.5–7765.4)	42	5428.9 (1715.8–14 213.2)	13	3909.5 (2470.2–10 141.8)	14	232.0 (186.4–444.0)
Fibrinogen, mg/dL	18	288.2 (159.4–347.6)	50	247.6 (171.8–333.5)	42	285.8 (234.7–332.6)	13	231.8 (173.3–336.0)	14	321.5 (282.2–401.0)
Triglyceride, mg/dL	18	85.5 (60.7–101.7)	50	107.7 (92.0–140.8)	42	133.4 (108.8–171.3)	12	149.9 (90.7–182.8)	14	204.8 (161.5–283.2)
Soluble CD25, ng/mL	18	0.8 (0.6–1.1)	50	1.1 (0.7–1.5)	42	1.2 (1.0–1.7)	13	1.2 (1.1–1.5)	14	0.5 (0.4–0.7)
Soluble CD163, ng/mL	18	367.1 (306.8–492.5)	50	408.5 (296.1–513.1)	42	411.4 (272.6–641.7)	13	460.9 (359.4–692.3)	14	434.5 (333.2–514.9)
IL-18, ng/mL	18	244.5 (163.5–312.0)	50	301.5 (231.7–366.0)	42	309.0 (207.0–384.8)	13	252.0 (213.0–300.0)	14	199.5 (167.2–385.5)
IL-1Ra, ng/mL	16	10.0 (5.9–12.0)	47	4.2 (2.3–10.4)	42	2.5 (1.5–3.5)	12	1.8 (1.6–2.8)	14	0.7 (0.5–1.0)

Data are presented as median (interquartile range) unless otherwise indicated.

Abbreviation: IL, interleukin.

### NK Cell Phenotype and Disease Severity

**Figure 2.**
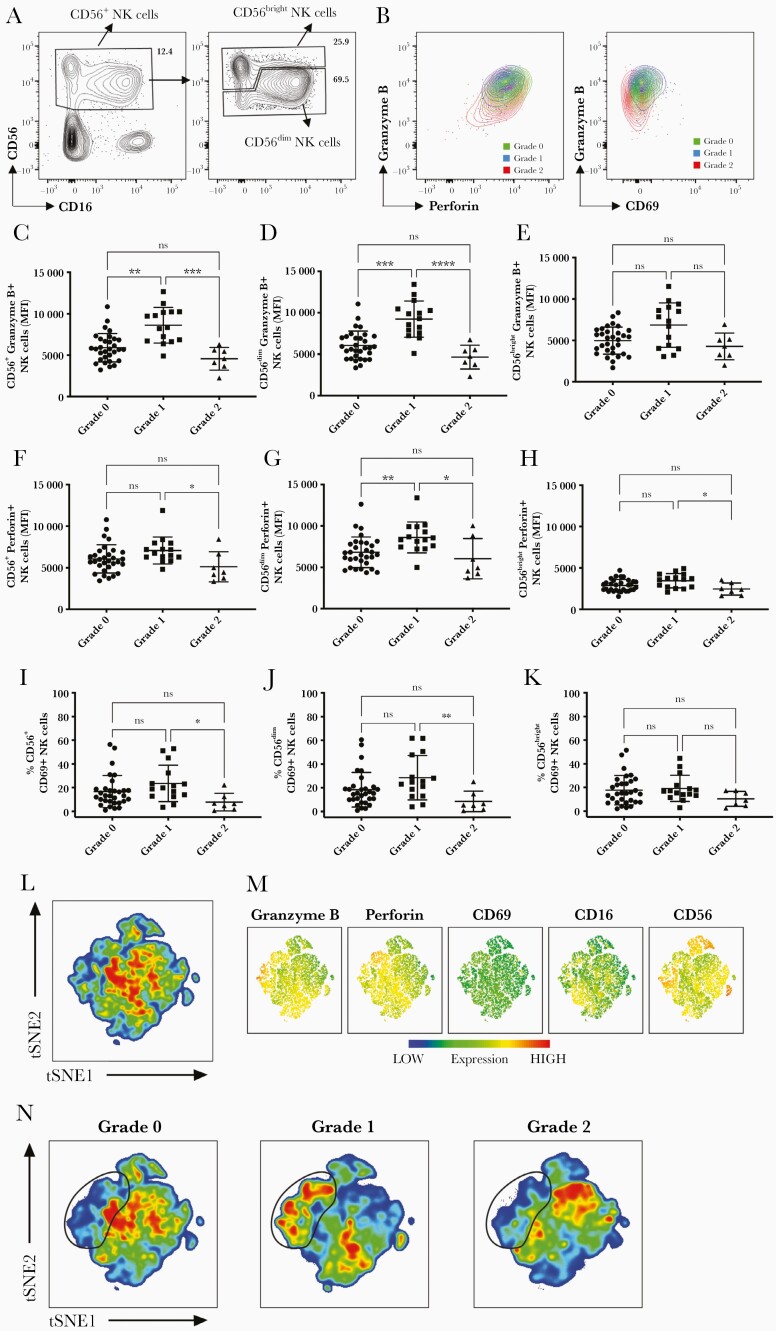
Natural killer (NK) cell phenotype. *A*, Identification of total CD56^+^, CD56^dim^, and CD56^bright^ NK cells based on CD56 and CD16 expression shown for a representative patient. Left plot is gated on live, CD14^–^CD3^–^ cells; right plot is gated on total CD56^+^ NK cells. *B*, Plots showing granzyme B and perforin (left) or CD69 (right) expression in 3 representative patients with grade 0, 1, and 2. *C–E*, Granzyme B expression depicted as mean fluorescence intensity (MFI) in CD56^+^ (*C*), CD56^dim^ (*D*), and CD56^bright^ NK cells (*E*) from grade 0, 1, and 2 patients. *F–H*, Perforin expression depicted as MFI in CD56^+^ (*F*), CD56^dim^ (*G*), and CD56^bright^ NK cells (*H*). *I–K*, Percentages of CD69^+^ cells were expressed in CD56^+^ (*I*), CD56^dim^ (*J*), and CD56^bright^ NK cells (*K*). *L–N*, Live CD14^–^CD3^–^ cells were concatenated after downsampling and analyzed simultaneously by *t*-distributed stochastic neighbor embedding (tSNE) in all 50 patients (*L*); levels of expression of granzyme B, perforin, CD69, CD16, and CD56 are shown in *N* from blue to orange (low to high, respectively). tSNE maps from patients with grade 0, 1, and 2 are shown separately (left to right, respectively). A cluster of NK cells with high levels of expression of granzyme B and perforin is highlighted with a black line. All analysis included in this figure were performed at days 5–8 from illness onset (grade 0, 5.68 ± 1.05 days; grade 1, 5.33 ± 0.72 days; grade 2, 6.14 ± 1.34 days) for 50 patients (grade 0, n = 28; grade 1, n = 15; grade 2, n = 7). Statistics were calculated by Kruskal–Wallis test followed by Dunn multiple comparison test. *P < .05; **P < .01; ***P < .001; ****P < .0001; ns, not significant.

Our results show that the frequencies of CD56^+^ NK cells, as well as those of the CD56^dim^ and CD56^bright^ NK cell subsets, were similar in the peripheral blood of patients with different grades of disease severities (Supplementary Figure 1*A–C*); however, the expression of perforin and granzyme B was significantly decreased in NK cells from grade 2 compared to grade 1 patients (Figure 2A–H). Granzyme B and perforin expression, depicted as mean fluorescence intensity, were significantly reduced in, respectively, CD56^dim^ and CD56^+^ NK cells and CD56^dim^, CD56^bright^, and CD56^+^ NK cells (Figure 2C–H). CD56^+^ and CD56^dim^ NK cells from grade 1 patients displayed significantly increased levels of granzyme B compared to those from grade 0 patients and a trend toward increased expression of perforin, although the latter was significant higher only in CD56^dim^ NK cells from grade 1 vs grade 0. A similar trend was observed for the expression of the activation marker CD69, with frequencies of CD69^+^ NK cells being significantly reduced in CD56^+^ and CD56^dim^ but not CD56^bright^ NK cells from grade 2 vs grade 1 patients (Figure 2A, 2B, and 2I–K). The frequencies of CD69^+^CD56^+^ and CD56^dim^ NK cells that were proliferating, as assessed by expression of Ki67, was also decreased in grade 2 compared to grade 1 patients (Supplementary Figure 1*D–F*), while the total percentages of proliferating Ki67^+^ cells within the NK cell subsets was similar in patients with different disease severities (Supplementary Figure 1*G–I*).

To better visualize differences in NK cell populations in patients with grade 0, 1, and 2 severities, we simultaneously compared expression of the cytotoxic/activation markers in all 50 patients analyzed using tSNE, a dimensionality reduction algorithm. Based on the tSNE maps of patients with grade 0, 1, and 2 dengue, we observed the presence of a cluster of NK cells with high intensity of expression of perforin and granzyme B in grade 1 patients, which appears diminished in both grade 0 and grade 2 patients (Figure 2L–N), in line with data shown in Figure 2C–H.

In summary, using flow cytometry analysis, we observed a reduction in activated CD56^+^ and CD56^dim^ NK cells and reduced cytotoxic potential in grade 2 compared to grade 1 patients. The profiles for granzyme B, perforin, and CD69 expression displayed a similar “bell-like” shape with expression increasing from grade 0 to 1 and decreasing from grade 1 to 2.

### NK Cell Genetic Polymorphisms and Disease Severity

**Figure 3.**
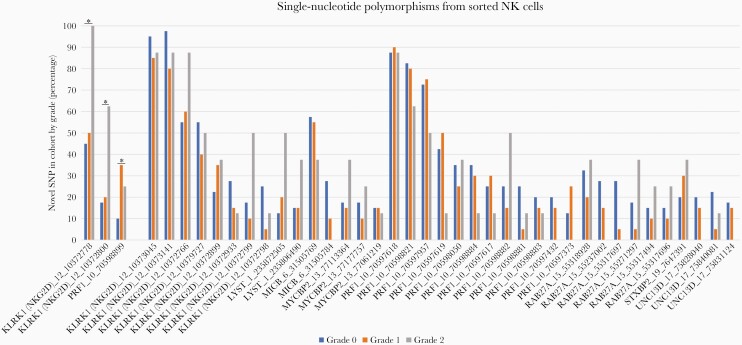
Detection of novel single-nucleotide polymorphisms (SNPs) from sorted natural killer (NK) cells in genes known to be involved in NK cell cytotoxic pathway. Presence of SNPs in the cohort is shown by clinical grade: grade 0 (blue), grade 1 (orange), and grade 2 (gray). Asterisks indicate positions that varied significantly between clinical grades.

We did not find any of the known HLH/MAS SNPs to be associated with dengue severity in this cohort (Supplementary Table 2). However, we did identify 3 previously unreported SNPs in the genes involved in NK cell function to be associated with more severe disease (grade 2 leakage) compared to patients with grade 1 and no leak (Figure 3). Two of the SNPs associated with severe dengue were located in the NK cell lectin-like receptor K1 gene (*KLRK1*). This gene is located on chromosome 12 within the NK group 2 (NKG2) complex.

Another SNP associated with disease severity was in the *PRF1* gene, which encodes perforin 1. *PRF1* mutations have been known to cause decreased or absent perforin protein expression on the surface of cytotoxic cells, which prevents cytotoxic immune cells and NK cells from destroying their target cells [23]. These known *PRF1* gene SNPs were not detected in this cohort. However, a novel *PRF1* SNP at position Chr10:70598899 (rs885821) was more prevalent in patients with grade 1 and 2 dengue compared to those with grade 0 (35%, 25%, and 10%, respectively). This is a synonymous SNP located in the promoter flanking region, which is where the transcription starts containing the promoter, enhancers, or other protein-binding sites.

## DISCUSSION

We have shown that features of MAS/hyperinflammation are associated with dengue disease severity, including inflammatory biomarkers, impaired NK cell activation/cytotoxic potential and novel NK cell gene polymorphisms. Patients with severe plasma leakage (the most common complication in dengue) have higher levels of hyperinflammatory/MAS biomarkers, including peak ferritin, triglycerides, and higher H-scores. These severe patients also had a lower percentage of activated NK cells and lower expression of the cytotoxic granules perforin and granzyme (Figure 2), as well as specific SNPs in genes involved in NK cell cytolytic function (*KLRK1* and *PRF1*). Other studies have shown that markers of MAS are more prevalent in severe dengue; however, this is the first study to demonstrate the potential mechanisms underlying this dengue-associated MAS, with dysfunctional NK cells and related NK cell SNPs also showing an association with severe disease.

Patients with severe disease had higher H-scores and a higher proportion had ferritin levels >2000 ng/mL. High ferritin levels correlate with inflammatory activity, macrophage activation, and mortality, irrespective of underlying trigger [24]. Higher plasma concentrations of ferritin have been found to be associated with worse clinical outcomes in many other dengue studies [13, 14, 25]. As ferritin levels are easily measured in clinical settings, ferritin could be a useful parameter to routinely screen patients for MAS/hyperinflammatory phenotype and enrollment into potential future therapeutic trials. We have demonstrated that NK cell cytotoxic dysfunction (lower expression of perforin and granzyme B) is associated with more severe dengue disease and may underly the hyperinflammatory/MAS phenotype. CD56^dim^ NK cells display profound cytotoxic and cytokine-producing capabilities in the blood and play a key role in killing of virus-infected cells. CD56^bright^ NK cells exert important tissue immunoregulatory roles through production of cytokines and chemokines [26]. The release of perforin and granzyme B are critical steps in NK cell–mediated cytotoxicity as perforin forms pores in the cell membrane of target cells that enable the entry into the cell of the serine protease granzyme B [27]. Our previous work showed that during acute dengue infection, the large proportion of NK cells in the blood was CD56^dim^ [28]. With reduced NK cell cytotoxic capacity, uncontrolled macrophage expansion can occur, which augments and prolongs release of proinflammatory cytokines and downstream tissue damage. Previous studies have shown that NK cell cytotoxic ability correlates with dengue viral clearance and better clinical outcomes [29] and uncomplicated dengue infection associates with a robust NK cell response [28]. We did not observe significant differences in monocyte populations or blood-derived macrophages with M2 phenotype in this dataset (Supplementary Figure 2*A–D*), although the expression levels of membrane-bound CD163 in the most abundant CD163^+^CD206^–^ subset appeared higher in grade 2 compared to grade 0 (Supplementary Figure 2*E*), fitting with the raised soluble CD163 in grade 2 patients.

The NK cell profiles in Figure 2 demonstrate that the NK cell response increases appropriately with increasing disease from grade 0 to 1; however, in grade 2 patients, rather than continue to increase proportionate to disease severity, paradoxically, the NK responses are reduced to levels observed in grade 0, suggesting an impaired response in severe dengue. Due to the small blood volumes available, we were unable to perform functional assays to directly assess NK cell cytotoxicity.

Genetic polymorphisms in the genes responsible for NK cell cytotoxicity may underlie the NK cell phenotypic dysfunction and the associated hyperinflammatory syndromes, triggered by various infections. Some of these genes, especially those encoding perforin and proteins required for movement and fusion of perforin-containing granules, are well described in MAS/HLH [30]. Although we were unable to identify known MAS/HLH SNPs in our cohort, we describe previously unreported SNPs in the same genes, which could have the same effect of reducing the NK cell functional ability to lyse viral infected cells, leading to hyperinflammation and a clinical syndrome of MAS.

The novel SNPs were identified in the coding regions of the genes *KLRK1* and *PRF1*. *KLRK1* encodes NKG2D, an activating receptor expressed on most NK cells that regulates signaling of immune responses against tumors, virally infected cells, and organ transplantations [31]. The NKG2D receptor is a homodimer found on the surface of all NK cells, as well as CD8^+^ T cells. NKG2D is involved in various processes of recognition and elimination of infected cells through regulation of signaling through other receptors [32].

NK and T cells generally require a secondary signal before NKG2D is able to mediate a measurable effect [32]. Within the context of immune response to viral infections, virus-infected cells often increase the expression of surface ligands, enabling recognition by NK cell–activating receptors, including NKG2D and DNAM-1 [33]. Genetic variants in this gene are likely to have an impact on immune modulation through binding of regulatory elements.

Mutations in *PRF1* occur in up to 50% of patients with familial HLH [34, 35]. The SNP we identified (Rs885821) has been identified in other studies, but no reports so far identify a relationship between this SNP and MAS/HLH [34, 35]. This *PRF1* SNP could functionally cause the reduced phenotypic expression of perforin from NK cells, affecting the regulation of the immune system and ability of NK cells to lyse target cells.

Further support for a possible role of NK cells is provided by a genome-wide association study that identified an association between a polymorphism in MICB (an NK cell–activating ligand related to KLKR1) and dengue severity. We were not able to identify this polymorphism using RNA sequencing, as this SNP is in the promoter region; however, the related SNPs point toward the same mechanism of a dysfunctional NK cell cytotoxic capability.

There were several limitations to the study, including the small sample size, especially in the enrollment of patients with severe dengue. The patients enrolled in this study were mainly adults and from 1 referral hospital, making the results less generalizable. NK cell dysfunction and macrophage activation may lead to uncontrolled viral replication and higher viremias; however, we were not able to assess this association in this clinical study, as viremia was only measured at 1 late time-point (hospitalization), so we do not have information on peak viremias or viral kinetics.

These results do, however, serve to fill some of the large gaps in our understanding of the underlying mechanisms of hyperinflammation and MAS/HLH in dengue. Confirmation of these novel SNPs in a different cohort and with a larger sample size is now required.

In summary, we have demonstrated that a hyperinflammatory/MAS phenotype occurs in dengue and is associated with disease severity. We have provided critical mechanistic data for this observed MAS clinical phenotype, with detailed immunological and transcriptomic analysis, showing underlying dysfunctional NK cells and specific NK cell gene SNPs. These results not only facilitate identification of patients at the highest risk of severe dengue but also patients who could respond to targeted immunomodulatory therapies. Clinical trials of targeted therapeutic immunomodulation for patients with dengue-associated MAS are now warranted.

## Supplementary Data

Supplementary materials are available at *The Journal of Infectious Diseases* online. Supplementary materials consist of data provided by the author that are published to benefit the reader. The posted materials are not copyedited. The contents of all supplementary data are the sole responsibility of the authors. Questions or messages regarding errors should be addressed to the author.

jiac093_suppl_Supplementary_Appendix

## Notes

***Acknowledgments.*** We thank all the medical and nursing staff in the National Hospital for Tropical Diseases who helped with patient recruitment for the original clinical study.

***Author contributions.*** Conceptualization: S. Y., L. R., K.-W. C., O. S., and E. E. O. Immunology studies: K.-W. C., Y. S. L., and Z. N. B. H. Biomarker analysis: N. L. V. and H. T. L. D. RNA sequencing analysis: O. S., T. T. V., and B. P. Flow cytometry data analysis: M. G. Writing: All authors.

***Disclaimer.*** The funding sources had no role in study design; collection, analysis, or interpretation of data; writing of the report; or decision to submit the manuscript for publication.

***Financial support.*** This work was supported by the Young Investigator Research Grant (National Medical Research Council [NMRC]/OFYIRG17may066 to S. Y.); The Wellcome Trust (grant number 100562/Z/12/Z to S. Y.); Open Fund–Individual Research, NMRC, Singapore (grant number NMRC/OFIRG/0049/2017 to L. R.); and the Singapore Ministry of Education (https://www.moe.gov.sg, with a startup grant to O. S.).
