# Supplementary material for: Hyperinflammatory Syndrome, Natural Killer Cell Function, and Genetic Polymorphisms in the Pathogenesis of Severe Dengue
Source: J Infect Dis. 2022 Mar 10;226(8):1338–47. doi: 10.1093/infdis/jiac093 (PMC9574659; doi:10.1093/infdis/jiac093)
Supplement: jiac093_suppl_Supplementary_Appendix [file jiac093_suppl_supplementary_appendix.docx]

**Appendix 1. Methodology**

**Clinical Endpoint definition**

Patients with dengue were subdivided by plasma leakage grade as as following. Grade 2 (severe plasma leakage) was defined as a haemoconcentration percentage (Δ Haematocrit [HCT]) of >20% and/or shock or pleural effusion with respiratory compromise. Grade 1 (moderate plasma leakage) was defined as a ΔHCT of 15-20% and/or any sign of fluid accumulation on clinical examination or X-ray/ultrasound without shock or respiratory compromise. Grade 0 (no clinically significant plasma leakage) was defined as a ΔHCT of <15% and no sign of fluid accumulation on clinical examination or X-ray/ultrasound. The haemoconcentration percentage (ΔHCT) was defined as (peak HCT – baseline HCT)/baseline HCT x 100. Clinical examination and chest X-ray and/or ultrasound of the lungs and abdomen were performed to assess for extravascular fluid accumulation. In addition, the WHO 2009 classification was used as per guidelines. Patients were defined as having MAS/hyperinflammation if they had at least three of the following criteria during the disease course: (i) fever ≥38.5°C; (ii) two line cytopaenia (platelet count <100×10^9^/l and neutrophil count <1×10^9^/l); (iii) hepatomegaly and/or AST >100 U/L; (iv) ferritin >5000 ng/ml; and (v) triglyceride >250 mg/dl or fibrinogen <150 mg/dl.

**Dengue Diagnositcs**

An NS1 test (Platelia enzyme-linked immunosorbent assay; BioRad), reverse transcription polymerase chain reaction (RT-PCR) and commercial IgM serology assays (Capture ELISA, Panbio) were used to confirm dengue virus (DENV) infection. Patients were defined as having laboratory confirmed dengue if RT-PCR, NS1 antigen or DENV IgM assays were positive at enrolment, or if there was IgM seroconversion between paired serum samples.

**PBMC preparation**

PBMCs were separated using density gradient sedimentation with ficoll/Hypaque and stored in liquid nitrogen. For further analysis they were shipped on liquid nitrogen to Duke-NUS Medical School in Singapore and stored in a liquid nitrogen tank. Cryopreserved PBMCs were thawed and washed in PBS before staining with a Fixable LIVE/DEAD Blue dead cell stain kit (Life Technologies) for 10 min at room temperature, for the exclusion of dead cells. PBMCs were then washed and stained on ice for 20 minutes with antibodies targeting cell surface markers, diluted in PBS (Hyclone) 1% BSA (Sigma Aldrich) with 0.1% sodium azide. Cells were then fixed for 45 min in eBioscience Foxp3/Transcription factor fixation/permeabilization buffer (Invitrogen) and intracellular staining was performed for detection of Ki67, perforin and granzyme A/B using eBioscience Foxp3/Transcription factor permeabilization buffer (Invitrogen) for 30 min on ice. Cells were acquired on a BD LSR Fortessa cytometer (Flow cytometry facility SingHealth/Duke-NUS). Samples were analyzed after compensation of spectral overlap between fluorophores using single stained controls and FlowJo (Version 10.5.3, FlowJo LLC). Data was analyzed by FlowJo version 10.5.3 and Prism Graph Pad version 9.1c.

**Cell sorting and RNA extraction**

Thawed PBMCs from 68 patients (no adequate sample for 1 patient) were stained with Fixable LIVE/DEAD Blue dead cell stain kit, followed by cell surface staining with fluorochrome conjugated antibodies listed in Table S1-B. Cells were surface stained in PBS supplemented with 1% BSA, and NK populations were purified by cell sorting on a BD FACSAria III cell sorter (Flow cytometry facility SingHealth/Duke-NUS). Single stained controls were used to compensate for spectral overlaps between fluorophores and NK cells were sorted from PBMCs (gating strategy shown in Fig. S3) directly lysed in the RLT lysis buffer (Qiagen) supplemented with beta-mercaptoethanol (Sigma). Lysed cells were immediately frozen on dry ice and kept in -80°C for RNA extraction. RNA extraction was performed using AllPrep DNA/RNA Micro Kit (Qiagen). Cell sorting purity for NK cells ranged from 90.1-97.2%.

**Supplementary Table 1-A.** List of antibodies used for the flow cytometry analysis

| Antibody  (anti-human) | Clone | Brand | Catalog number | Dilution (ul) in 50ul |
| --- | --- | --- | --- | --- |
| CD3 V500 | UCHT1 | BD | 561416 | 3 |
| CD14 BUV395 | MφP9 | BD | 563561 | 2.5 |
| CD16 BV711 | 3G8 | Biolegend | 302044 | 3.5 |
| CD56 PE CF594 | NCAM 16.2 | BD | 564849 | 0.25 |
| CD69 BV785 | FN50 | Biolegend | 310932 | 5 |
| CD163 BV605 | GHI/61 | Biolegend | 333616 | 3.5 |
| CD206 AF488 | 15-2 | Biolegend | 321114 | 5 |
| HLA-DR APC-Cy™7 | L243 | BD | 335796 | 3 |
| Ki67 BV421 | Ki-67 | Biolegend | 350506 | 5 |
| Perforin PerCP/Cyanine5.5 | B-D48 | Biolegend | 353314 | 3 |
| Granzyme B PE Cy7 | QA16A02 | Biolegend | 372214 | 3 |

**Supplementary Table 1-B.** List of antibodies used for the cell sorting panel

| Antibody  (anti-human) | Clone | Brand | Catalog number | Dilution (ul) in 50ul |
| --- | --- | --- | --- | --- |
| CD3 V450 | UCHT1 | BD | 560365 | 2 |
| CD14 PE-Cy™7 | M5E2 | BD | 560774 | 1 |
| CD19 PerCP/Cyanine5.5 | HIB19 | Biolegend | 557742 | 1 |
| CD56 Alexa Fluor® 488 | HCD56 | Biolegend | 302230 | 3 |

**Supplementary Table 2.** Genetic polymorphisms in genes known to be associated with viral-associated macrophage activation syndrome (MAS)

The single nucleotide polymorphisms (SNPs) in genes known to be associated with viral infection and MAS (PRF1, STX11, STXBP2, UNC13D, LYST and RAB27A) listed in ClinVar (https://www.ncbi.nlm.nih.gov/clinvar/) were converted to Ensembl coordinates and searched for their presence in our cohort. With the exception of ENSG00000143669.13 and ENSG00000069974.15 which were each present in one person, these variants were not identified in our cohort.

**Appendix 2. Additional results**

**Supplementary Figure 1.** NK cell frequencies and phenotype

**
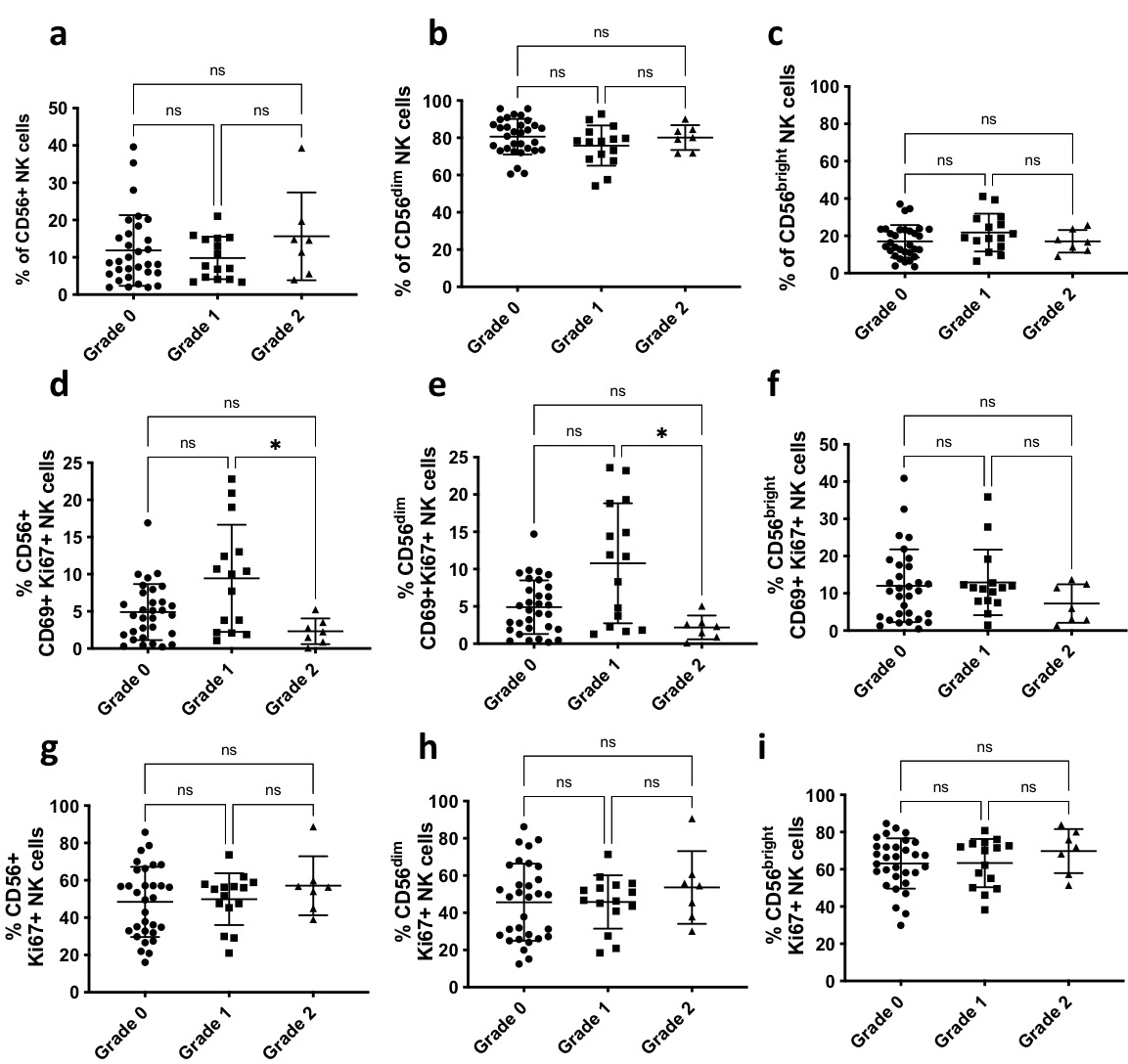
**

Frequencies of CD56+ (a), CD56^dim^ (b), and CD56^bright^ (c) NK cells in patients with grade 0, 1 and 2 severities. Percentages of CD69+ Ki67+ cells (d-f) and total Ki67+ (g-i) in CD56+ (d, g), CD56^dim^ (e, h), and CD56^bright^ (f, i) cells in patients with grade 0, 1 and 2 severities.

**Supplementary Figure 2.** Monocytes and M2 macrophages

**
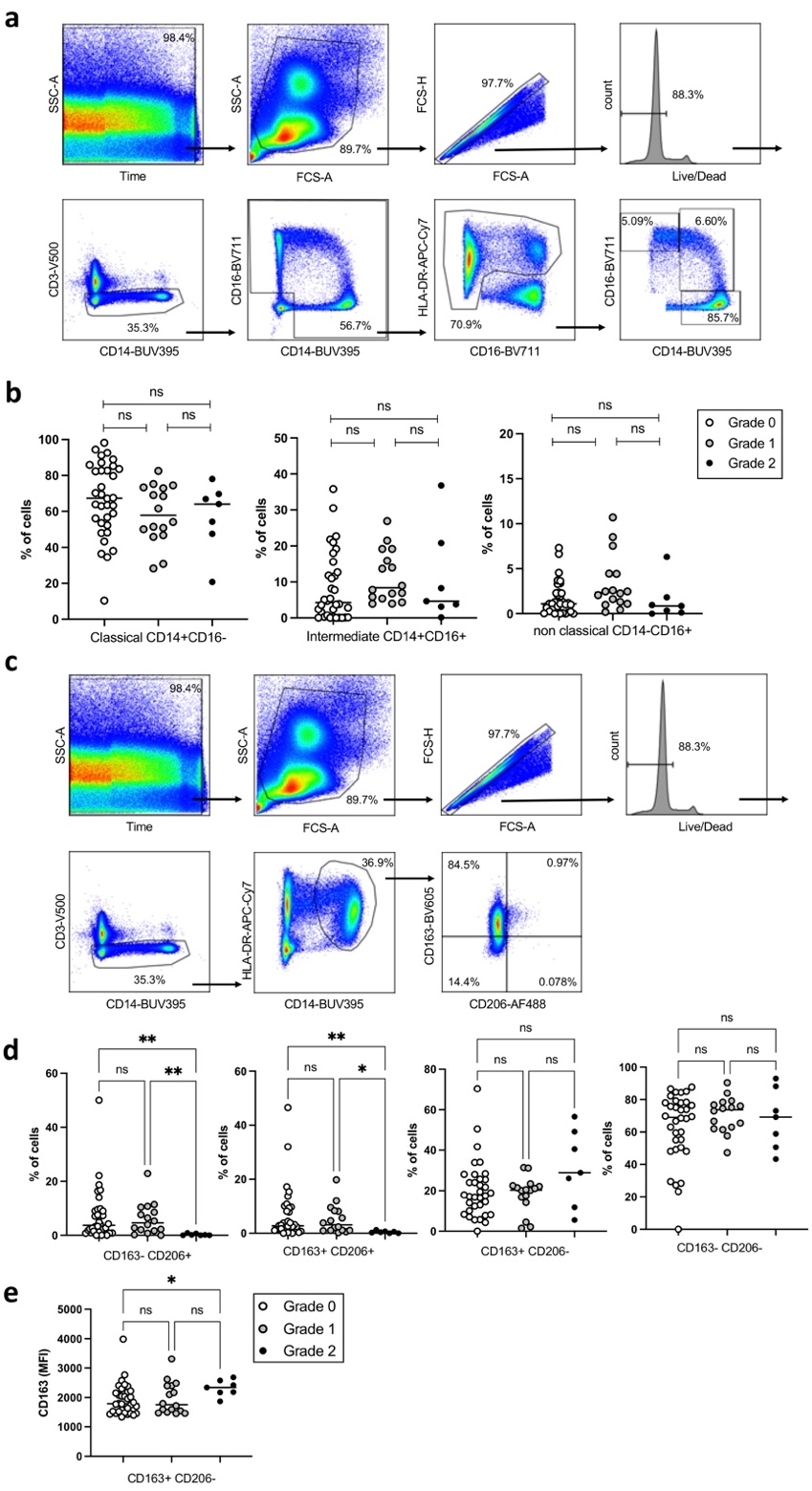
**

**(a, b) Monocyte populations**. The gating strategy to identify monocyte populations is shown for a representative donor in (a). The percentages of classical (CD14+ CD16-), non-classical (CD16+ CD14-) and intermediate (CD14+ CD16+) monocytes calculated from the “true monocyte” gate are shown in (b). **(c-e) M2 Macrophages**. The gating strategy to identify populations of cells expressing CD163 and/or CD205 is shown in (c). The percentages of cells from each subpopulation defined by CD163/CD206 expression within HLA-DR+ CD14+ cells are shown in (d). The Mean Fluorescence Intensity (MFI) of CD163 expression in the most abundant CD163+ CD206- subset in peripheral blood is shown in (e). Statistics in b, d and e are calculated using a non-parametric Kruskal-Wallis test followed by Dunn’s multiple comparison test.

**Supplementary Figure 3.** Gating strategy


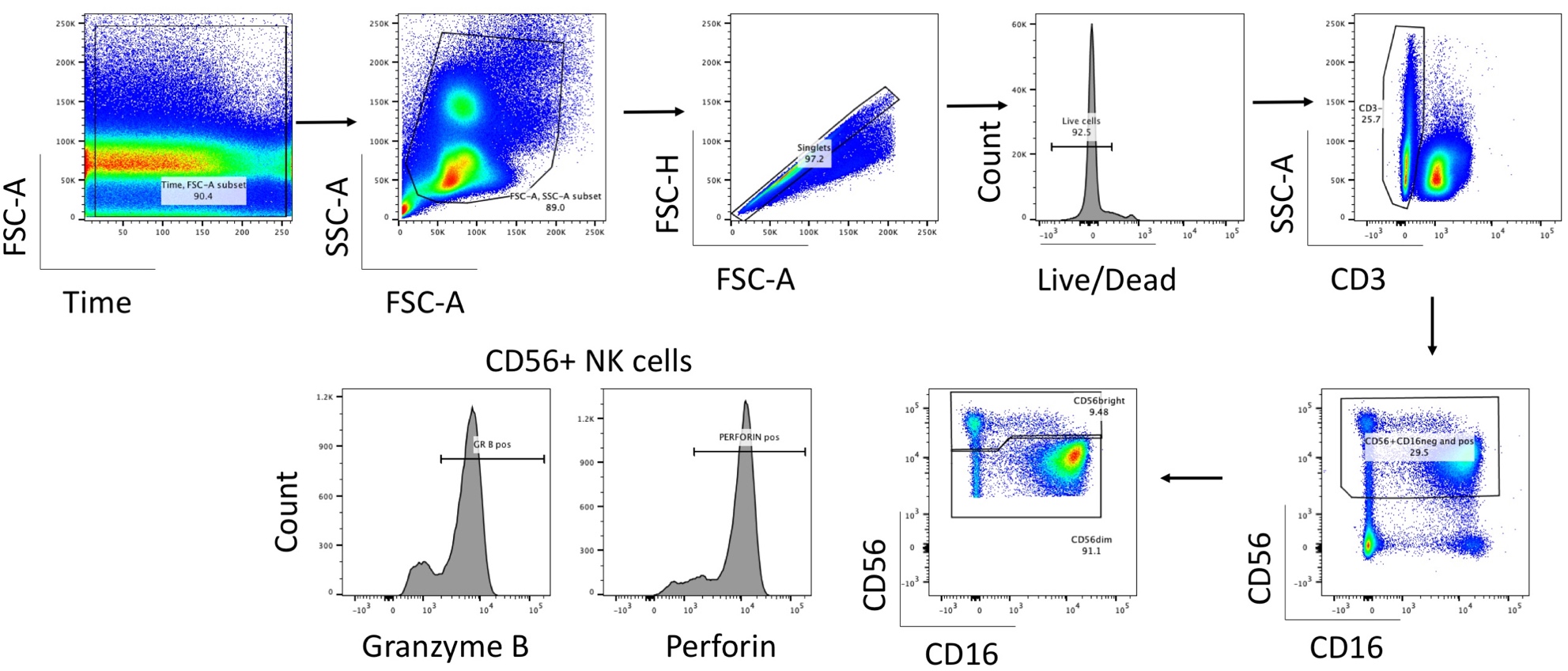


NK cell gating strategy for the flow cytometry and cell sorting experiments showed for a representative donor. For the flow cytometry analysis: cells were gated based on acquisition time (time/FSC-A) and FSC-A/SSC-A. Gates were subsequently applied to identify single cells (FSC-A/FSC-H), live cells and CD14- cells. CD3- cells were then gated for the identification of NK cells based on CD56 and CD16 expression. Mean Fluorescence Intensity (MFI) values for granzyme B and perforin were calculated on the granzyme B+ and perforin+ gates (shown for CD56+ NK cells), respectively. For cell sorting: cells were gated as shown from the FSC-A/SSC-A gate. Total CD56+ NK cells were sorted directly into lysis buffer.
